# Efficacy and safety of ruxolitinib in steroid-refractory graft-versus-host disease: A meta-analysis

**DOI:** 10.3389/fimmu.2022.954268

**Published:** 2022-08-04

**Authors:** Shuang Fan, Wen-Xuan Huo, Yang Yang, Meng-Zhu Shen, Xiao-Dong Mo

**Affiliations:** ^1^ Peking University People’s Hospital, Peking University Institute of Hematology, National Clinical Research Center for Hematologic Disease, Beijing Key Laboratory of Hematopoietic Stem Cell Transplantation, Beijing, China; ^2^ Research Unit of Key Technique for Diagnosis and Treatments of Hematologic Malignancies, Chinese Academy of Medical Sciences, 2019RU029, Beijing, China

**Keywords:** acute graft-versus-host disease, chronic graft-versus-host disease, ruxolitinib, steroid-refractory, meta-analysis

## Abstract

Ruxolitinib is an important treatment for steroid refractory graft-versus-host disease (SR-GVHD). Therefore, we reported the updated results of a systematic review and meta-analysis of ruxolitinib as treatment for SR-GVHD. In addition, we wanted to compare the efficacy and safety between children and adults with SR-GVHD. Overall response rate (ORR) after ruxolitinib treatment was chosen as the primary end point. Complete response rate (CRR), infection, myelosuppression, and overall survival (OS) were chosen as secondary end points. A total of 37 studies were included in this meta-analysis, and 1,580 patients were enrolled. ORR at any time after ruxolitinib treatment was 0.77 [95% confidence interval (CI): 0.68–0.84] and 0.78 (95% CI: 0.74–0.81), respectively, for SR-aGVHD and SR-cGVHD. CRR at any time after ruxolitinib treatment was 0.49 (95% CI: 0.40–0.57) and 0.15 (95% CI: 0.10–0.23), respectively, for SR-aGVHD and SR-cGVHD. The ORRs at any time after treatment was highest in mouth SR-cGVHD, followed by skin, gut, joints and fascia, liver, eyes, esophagus, and lung SR-cGVHD. The incidence rate of infections after ruxolitinib treatment was 0.61 (95% CI: 0.45–0.76) and 0.47 (95% CI: 0.31–0.63), respectively, for SR-aGVHD and SR-cGVHD. The incidence rates of overall (grades I–IV) and severe (grades III–IV) cytopenia were 53.2% (95% CI: 16.0%–90.4%) and 31.0% (95% CI: 0.0–100.0%), respectively, for SR-aGVHD, and were 28.8% (95% CI:13.0%–44.6%) and 10.4% (95% CI: 0.0–27.9%), respectively, for SR-cGVHD. The probability rate of OS at 6 months after treatment was 63.9% (95% CI: 52.5%–75.2%) for SR-aGVHD. The probability rates of OS at 6 months, 1 year, and 2 years after treatment were 95% (95% CI: 79.5%–100.0%), 78.7% (95% CI: 67.2%–90.1%), and 75.3% (95% CI: 68.0%–82.7%), respectively, for SR-cGVHD. The ORR, CRR, infection events, and myelosuppression were all comparable between children and adults with SR-GVHD. In summary, this study suggests that ruxolitinib is an effective and safe treatment for SR-GVHD, and both children and adults with SR-GVHD could benefit from ruxolitinib treatment.

## Introduction

Allogeneic hematopoietic stem cell transplantation (HSCT) is one of the most important treatments for patients with hematological malignancies and non-malignant disease (1). However, graft-versus-host disease (GVHD) is still a common complication. It can severely influence the quality of life (2–5) and is an important cause of transplant-related mortality (6, 7). Corticosteroid is the first-line treatment for GVHD, but the response rate was approximate 50% (8), and a significant number of patients will experience steroid-refractory GVHD (SR-GVHD) (9). Thus far, there is no effective treatment for patients with SR-GVHD (10), and their survival rate is poor (11).

Ruxolitinib is a potent and selective oral inhibitor of Janus kinase (JAK) 1 and JAK2 and is an important treatment for myeloproliferative neoplasms (12). In addition, JAKs are well positioned to regulate GVHD. A variety of cytokines, which signal through the JAK/STAT pathways, play a critical role in regulation of the proliferation and activation on immune cell types that are important for GVHD pathogenesis (13). Recently, ruxolitinib is under investigation for the treatment of SR-GVHD, and it has been reported to be an important treatment for SR-GVHD (14–16). Ruxolitinib has been approved for the treatment of SR acute GVHD (aGVHD) and chronic GVHD (cGVHD) by the Unite States Food and Drug Administration in 2019 and 2021, respectively (17, 18).

Thus far, there are many clinical studies focused on ruxolitinib for SR-GVHD treatment, and Li et al. (19) had conducted a meta-analysis for ruxolitinib as the treatment for SR-GVHD in adults; however, only studies published before January 2019 were enrolled. Since 2019, many important research studies for ruxolitinib in SR-GVHD have been published (15, 16, 20–28). In addition, this meta-analysis did not include children, whereas several studies focused on ruxolitinib for treatment of SR-GVHD in children have been published since 2019 (29–32). No systematic review was designed to compare the efficacy of ruxolitinib for SR-GVHD treatment between children and adults.

Therefore, we reported the updated results of a systematic review and meta-analysis of ruxolitinib as treatment for SR-GVHD. In addition, we wanted to compare the efficacy and safety between children and adults with SR-GVHD.

## Methods

### Inclusion criteria

The inclusion criteria were as follows: (1) patients of any race, any sex, and all ages; (2) those diagnosed with SR-GVHD (i.e., aGVHD or cGVHD) after HSCT; and (3) those using ruxolitinib as the treatment for SR-GVHD. Reviews, duplicates, and conference abstracts were excluded. While assessing multiple reports from the same study, we selected the report containing more information or with a longer follow-up.

### Search strategy

A literature search was conducted following the Preferred Reporting Items for Systematic Reviews and Meta-analyses statement (33). The PubMed and Embase databases were searched, with the search strategy following the Population (patients with steroid refractory GVHD), Intervention (ruxolitinib), Outcomes [overall response rate (ORR), complete response rate (CRR), infection, myelosuppression, and overall survival (OS)], and Study framework (retrospective, prospective non-randomized, and randomized trials) (34): [(Glucocorticoid-Refractory) OR (steroid refractory) OR (steroid-refractory) OR (steroid resistant) OR (steroid-resistant) OR (corticosteroid refractory) OR (corticosteroid-refractory)] AND [(acute graft versus host disease) OR aGVHD OR cGVHD OR (chronic graft versus host disease) OR (graft versus host reaction)] AND [(Ruxolitinib) OR (Janus Kinase Inhibitors) OR (JAK Kinases Inhibitors) OR (jak kinases Inhibitors)].

### Data extraction and outcomes

All data were independently extracted by two reviewers (Wen-Xuan Huo and Yang Yang) to ensure accuracy. Information on the following was extracted: study characteristics (e.g., study framework, first author, publish year, and follow-up period), patients (e.g., number, age, gender, and disease characteristics), transplantation (e.g., conditioning regimen, graft source, and type of transplant), GVHD (e.g., type, organ involved, and grade), ruxolitinib (e.g., dose, intervention time, and duration of treatment), and outcome parameters during the follow-up period.

The ORR after ruxolitinib treatment was chosen as the primary end point. The CRR, infection, myelosuppression, and OS were chosen as secondary end points. In addition, the response rates at 28 days and at 24 weeks after ruxolitinib were assessed in the analysis of SR-aGVHD and SR-cGVHD, respectively. Missing data were documented as “not available (NA)”. All data were extracted according to the Cochrane Handbook for Systematic Reviews of Interventions (35).

### Statistical analysis

The “meta” package version 4.16-2 (36) was used to perform the meta-analysis. Statistical heterogeneity among studies was assessed using the I^2^ statistics and Cochran Q-test. The random-effects model was adopted, with the heterogeneity test showing I^2^ > 50% and P < 0.10. The subgroup comparison of adults and children was also conducted. The null hypothesis was set to no difference. A *P*-value < 0.05 was considered statistically significant to reject the null hypothesis. The results were analyzed by the boxplot using “ggplot2” package version 3.3.5 (37).

## Results

### Included studies

**Figure 1:**
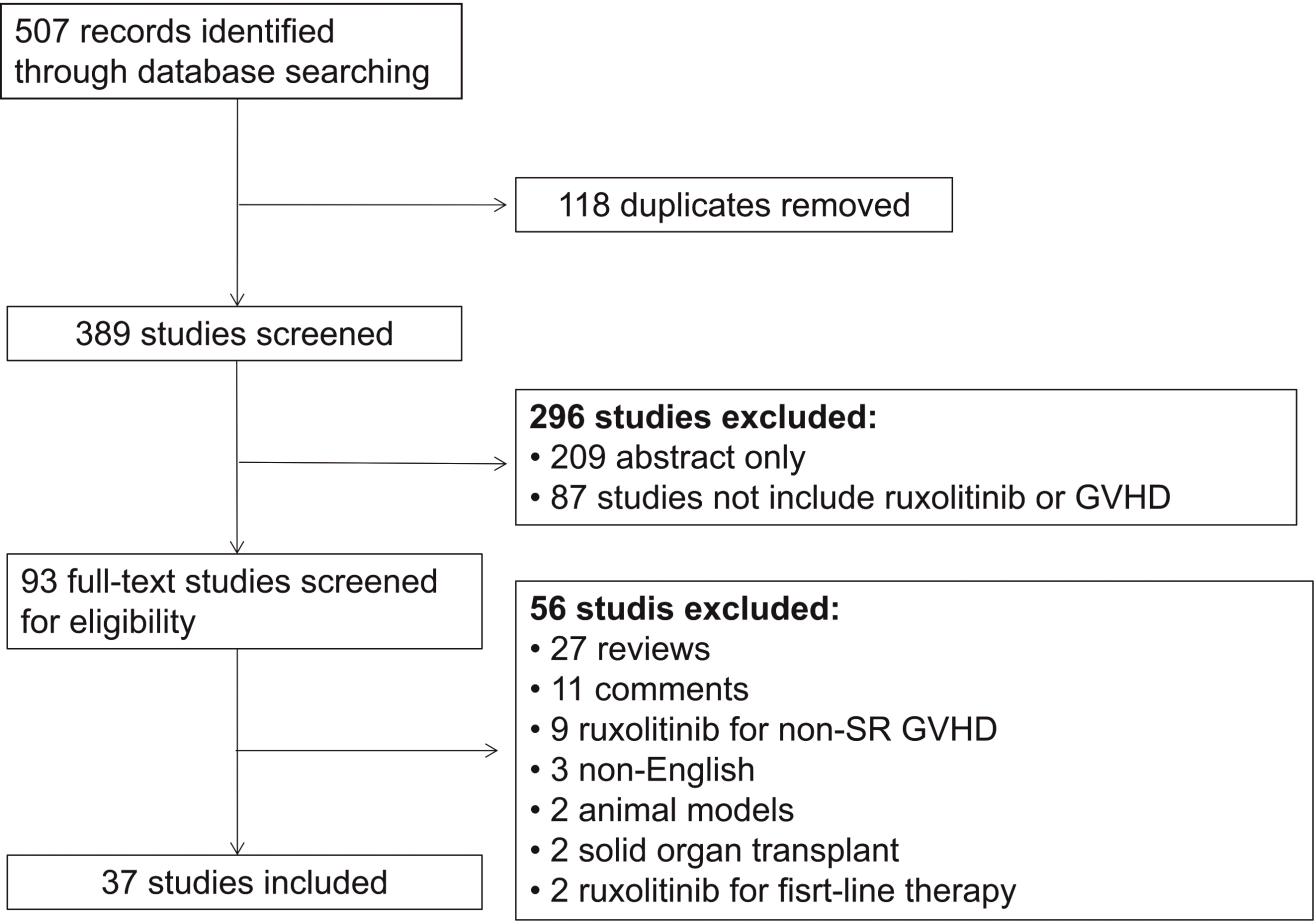
Selection scheme of studies. SR-GVHD, steroid-refractory graft-versus-host disease.

A total of 37 studies were included in this meta-analysis, and 1,580 patients were enrolled (**Tables 1**–**4** and **Figure 1**). **Table 5** summarized the studies for every subgroup analysis.

**Table 1:** Characteristics of 24 included studies (SR-aGVHD)^*^.

Studies	Median age/year (range)	HLA matching (*n*)				SR-aGVHD grade (*n*)				Median time from SR-aGVHD diagnosis to the application of ruxolitinib/day (range)
		MRD	mMRD	MUD	mMUD	I	II	III	IV	
Zeiser, 2020	52.5 (12–73)	NA	NA	NA	NA	2	50	68	30	NA
Modemann, 2020	58.5 (21–73)	3	0	9	6	0	0	9	9	87 (35–257)
Lancman, 2018	58 (33–61)	NA	NA	NA	NA	1	3	NA	NA	NA
Assouan, 2017	52 (26–65)	5	NA	5	NA	NA	NA	6	NA	14
Wei, 2021	30 (11–56)	5	NA	NA	NA	NA	9	8	6	5 (1–79)
Leung, 2022	38 (19–63)	NA	NA	NA	NA	0	13	4	5	NA
Moiseev, 2020	17 (1–67)	2	NA	19	NA	0	11	10	11	16 (5–113)
Jagasia, 2020	58 (18–73)	18	11	27	10	0	23	34	14	NA
Biliński, 2021	53.5 (22–66)	2	NA	2	NA	NA	NA	NA	4	NA
Liu, 2021	29 (13–63)	NA	NA	NA	NA	0	7	22	11	NA
Laisne, 2020	4.3 (0.4–14.5)	NA	NA	NA	NA	0	7	13	9	91 (17–518)
González, 2018	11 (5–18)	NA	NA	NA	NA	0	0	4	9	9
Maldonado, 2017	51 (28–56)	1	NA	NA	NA	NA	NA	3	NA	NA
Maldonado, 2021	32 (26–48)	NA	NA	NA	NA	NA	NA	2	7	NA
Meng, 2019	23.5 (8–38)	12	NA	NA	NA	0	6	5	1	NA
Mozo, 2021	8.6 (0.8–18.1)	3	NA	3	NA	NA	NA	2	6	NA
Khandelwal, 2017	8.5 (1.6–16.5)	1	NA	12	NA	NA	2	9	2	147 (55–538)
Zeiser, 2015	51 (21–75)	13	NA	15	NA	NA	NA	NA	NA	NA
Abedin, 2019	59 (46–70)	6	2	10	1	NA	3	13	3	21 (3–162)
Dang, 2020	35 (19–55)	8	2	NA	NA	NA	NA	9	NA	NA
Uygun, 2020	NA	4	NA	17	NA	NA	2	4	7	28 (7–231)
Gómez, 2019	51 (0–73)	33	NA	39	NA	NA	3	20	NA	NA
Toama, 2020	55 (27–72)	7	NA	24	NA	NA	NA	NA	NA	17 (2–280)
Zhao, 2020	29 (14–62)	5	55	4	NA	NA	NA	22	42	8 (3–89)

HLA, human leukocyte antigen; mMRD, mismatched related donor; MRD, matched related donor; mMUD, mismatched unrelated donor; MUD, matched unrelated donor; NA, not available; SR-aGVHD, steroid-refractory acute graft-versus-host disease.^*^Thirteen studies included both SR-aGVHD and SR-cGVHD analysis.

**Table 2:** Characteristics of 26 included studies (SR-cGVHD)^*^.

Studies	Median age/year (range)	HLA matching (*n*)				SR-cGVHD grade (*n*)			Median time from SR-cGVHD diagnosis to the application of ruxolitinib/day (range)
		MRD	mMRD	MUD	mMUD	Mild	Moderate	Severe	
Lancman, 2018	52 (38–71)	NA	NA	NA	NA	NA	NA	NA	NA
Hurabielle, 2017	47 (21–67)	4	NA	8	NA	NA	NA	NA	NA
Maas-Bauer, 2020	49 (28–70)	NA	NA	NA	NA	NA	NA	13	NA
Zeiser, 2022	49 (13–73)	91	NA	76	NA	1	67	97	NA
Ferreira, 2021	54 (23–73)	18	NA	8	NA	0	23	12	510 (30–2130)
Ferreira, 2018	46.5 (23–68)	10	NA	NA	NA	0	11	9	480
Wei, 2021	31 (11–54)	19	NA	1	NA	2	10	20	17 (7–1239)
Wang, 2021	35 (13–63)	27	NA	2	41	23	38	9	NA
Leung, 2022	33 (21–64)	NA	NA	NA	NA	7	15	7	NA
Moiseev, 2020	21 (2–62)	7	NA	30	NA	0	6	37	376 (28–3219)
González, 2018	11 (5–18)	NA	NA	NA	NA	1	1	7	540
Kaurinovic, 2022	60 (26–76)	NA	NA	NA	NA	10	35	8	NA
Maldonado, 2017	36 (26–52)	1	NA	2	NA	NA	1	4	180 (90–540)
Maldonado, 2021	38.5 (22–59)	NA	NA	NA	NA	2	1	5	NA
Schoettler, 2019	10 (7–21)	NA	NA	5	NA	NA	1	4	120 (75–720)
Modi, 2018	49 (21–77)	15	NA	10	16	3	8	35	NA
Mozo, 2021	12 (2.1–16)	1	NA	8	NA	NA	8	4	NA
Zeiser, 2015	55 (22–74)	9	NA	17	NA	NA	6	35	NA
Abedin, 2019	59 (45–70)	11	1	12	0	NA	16	8	NA
Redondo, 2022	49 (18–72)	27	NA	19	2	1	29	18	150 (18–630)
Dang, 2020	30 (14–55)	15	8	5	NA	NA	24	NA	NA
Uygun, 2020	NA	NA	NA	NA	NA	NA	2	13	840 (210–1560)
Gómez, 2019	NA	NA	NA	NA	NA	0	28	28	NA
Wu, 2021	31 (17–56)	9	NA	NA	NA	NA	14	27	330 (18–2,157)
Xue, 2021	45 (19–71)	10	14	12	NA	NA	9	27	654 (69–4,482)
Zhao,2021	27 (15–54)	8	21	1	NA	NA	6	24	125 (27–1,598)

HLA, human leukocyte antigen; mMRD, mismatched related donor; MRD, matched related donor; mMUD, mismatched unrelated donor; MUD, matched unrelated donor; NA, not available; SR-cGVHD, steroid-refractory chronic graft-versus-host disease. ^*^Thirteen studies included both SR-aGVHD and SR-cGVHD analysis.

**Table 3:** Characteristics of therapeutic response for 24 included studies (SR-aGVHD)^*^.

Studies	Study design	N	Response/event (n)				cGVHD incidence (%)	6-month overall survival	Median follow-up (months)
			ORR	ORR at 28 days	CRR	CRR at 28 days			
Zeiser, 2020	RCT	154	NA	96	NA	53	NA	NA	5.04
Modemann, 2020	Retrospective	18	10	NA	8	NA	66.70	NA	NA
Lancman, 2018	Case report	4	NA	NA	3	2	NA	0.75	NA
Assouan, 2017	Retrospective	10	NA	NA	5	NA	NA	0.70	4.47
Wei, 2021	Retrospective	23	20	NA	13	NA	21.74	NA	14.43
Leung, 2022	Retrospective	26	19	19	15	8	11.54	NA	NA
Moiseev, 2020	Prospective	32	24	NA	20	NA	37.50	NA	NA
Jagasia, 2020	Prospective	71	52	39	40	19	5.63	0.51	10.77
Biliński, 2021	Case report	4	NA	NA	2	NA	NA	NA	NA
Liu, 2021	Retrospective	40	34	NA	25	NA	7.50	0.57	8.20
Laisne, 2020	Retrospective	29	NA	NA	19	6	NA	NA	16.00
González, 2018	Prospective	13	NA	NA	4	NA	NA	NA	NA
Maldonado, 2017	Case report	3	3	NA	2	NA	NA	NA	24.00
Maldonado, 2021	Retrospective	9	7	NA	4	NA	NA	NA	NA
Meng, 2019	Retrospective	12	10	NA	7	NA	NA	NA	NA
Mozo, 2021	Retrospective	8	7	NA	3	NA	12.50	NA	NA
Khandelwal, 2017	Retrospective	13	5	NA	1	NA	NA	NA	NA
Zeiser, 2015	Retrospective	54	44	NA	25	NA	NA	0.79	6.18
Abedin, 2019	Retrospective	19	17	16	12	9	NA	NA	NA
Dang, 2020	Retrospective	10	10	NA	NA	NA	NA	NA	2.50
Uygun, 2020	Retrospective	13	11	NA	9	NA	NA	NA	NA
Gómez, 2019	Retrospective	23	16	NA	5	NA	NA	0.47	NA
Toama, 2020	Retrospective	36	14	NA	6	NA	NA	NA	NA
Zhao, 2020	Prospective	64	NA	56	NA	47	NA	0.68	15.50

CRR, complete response rate; NA, not available; ORR, overall response rate; RCT, randomized controlled trials; SR-aGVHD, steroid-refractory acute graft-versus-host disease; SR-cGVHD, steroid-refractory chronic graft-versus-host disease.^*^Thirteen studies included both SR-aGVHD and SR-cGVHD analysis.

**Table 4:** Characteristics of therapeutic response for 26 included studies (SR-cGVHD)^*^.

Studies	Study design	N	Response/event (n)				Overall survival			Median follow-up (months)
			ORR	ORR at 24 weeks	CRR	CRR at 24 weeks	6 months	12 months	24 months	
Lancman, 2018	Case report	4	NA	NA	1	NA	1.00	NA	NA	NA
Hurabielle, 2017	Retrospective	12	NA	NA	0	NA	NA	NA	NA	NA
Maas-Bauer, 2020	Retrospective	23	17	NA	2	NA	NA	NA	0.75	30.00
Zeiser, 2022	RCT	165	NA	82	NA	11	NA	NA	NA	NA
Ferreira, 2021	Retrospective	35	31	NA	9	NA	NA	NA	NA	43.00
Ferreira, 2018	Case report	20	15	NA	4	NA	NA	NA	NA	12.00
Wei, 2021	Retrospective	32	25	NA	8	NA	NA	NA	NA	16.50
Wang, 2021	Retrospective	70	NA	52	NA	34	NA	0.66	NA	13.37
Leung, 2022	Retrospective	31	NA	26	NA	16	NA	0.94	0.81	NA
Moiseev, 2020	Prospective	43	35	NA	9	NA	NA	NA	NA	NA
González, 2018	Prospective	9	NA	NA	2	NA	NA	NA	NA	NA
Kaurinovic, 2022	Retrospective	53	43	NA	28	NA	NA	NA	NA	20.00
Maldonado, 2017	Case report	5	5	NA	1	NA	NA	NA	NA	12.00
Maldonado, 2021	Retrospective	8	6	NA	4	NA	NA	NA	NA	NA
Schoettler, 2019	Case report	5	NA	NA	NA	NA	NA	NA	NA	24.00
Modi, 2018	Retrospective	46	NA	22	NA	5	NA	0.84	NA	NA
Mozo, 2021	Retrospective	12	11	NA	1	NA	NA	NA	0.76	NA
Zeiser, 2015	Retrospective	41	35	NA	3	NA	0.97	NA	NA	5.23
Abedin, 2019	Retrospective	24	20	NA	3	NA	NA	NA	NA	NA
Redondo, 2022	Retrospective	48	37	NA	7	NA	NA	NA	0.83	20.00
Dang, 2020	Retrospective	28	22	NA	NA	NA	NA	NA	NA	5.00
Uygun, 2020	Retrospective	15	13	NA	1	NA	NA	NA	NA	NA
Gómez, 2019	Retrospective	56	32	NA	2	NA	NA	0.81	NA	NA
Wu, 2021	Retrospective	41	NA	29	NA	15	0.88	0.66	NA	14.90
Xue, 2021	Retrospective	36	NA	16	NA	4	NA	0.81	0.74	NA
Zhao, 2021	Retrospective	30	20	17	3	NA	NA	NA	0.63	10.60

CRR, complete response rate; NA, not available; ORR, overall response rate; RCT, randomized controlled trials; SR-aGVHD, steroid-refractory acute graft-versus-host disease; SR-cGVHD, steroid-refractory chronic graft-versus-host disease.

^*^Thirteen studies included both SR-aGVHD and SR-cGVHD analysis.

**Table 5:** The number of studies included in the subgroup analysis.

Subgroup	Studies included, No. (%)	
	SR-aGVHD^1^	SR-cGVHD^1^
**ORR**		
At any time	**n = 17**	**n = 16**
Retrospective studies	15 (88.2)	15 (93.8)
Prospective unrandomized studies	2 (11.8)	1 (6.2)
RCT	NA	NA
At day 28	**n = 5**	NA
Retrospective studies	2 (40.0)	NA
Prospective unrandomized studies	2 (40.0)	NA
RCT	1 (20.0)	NA
At week 24	NA	**n = 7**
Retrospective studies	NA	6 (85.7)
Prospective unrandomized studies	NA	NA
RCT	NA	1 (14.3)
**CRR**		
At any time	**n = 21**	**n = 18**
Retrospective studies	18 (85.7)	16 (88.9)
Prospective unrandomized studies	3 (14.3)	2 (11.1)
RCT	NA	NA
At day 28	**n = 7**	NA
Retrospective studies	4 (57.1)	NA
Prospective unrandomized studies	2 (28.6)	NA
RCT	1 (14.3)	NA
At week 24	NA	**n = 6**
Retrospective studies	NA	5 (83.3)
Prospective unrandomized studies	NA	NA
RCT	NA	1 (16.7)
**Involved Organ Response**		
ORR**^2^**	**n = 9**	**n = 13**
Skin	8 (88.9)	13 (100.0)
Gut	9 (100.0)	11 (84.6)
Liver	8 (88.9)	9 (69.2)
Mouth	NA	8 (61.5)
Eye	NA	10 (76.9)
Lung	NA	12 (92.3)
Joints and fascia	NA	8 (61.5)
Esophagus	NA	1 (7.7)
CRR**^2^**	**n = 8**	**n = 11**
Skin	7 (87.5)	11 (100.0)
Gut	8 (100.0)	9 (81.8)
Liver	7 (87.5)	7 (63.6)
Mouth	NA	7 (63.6)
Eye	NA	7 (63.6)
Lung	NA	10 (90.9)
Joints and fascia	NA	7 (63.6)
Esophagus	NA	1 (9.1)
**Hematologic Toxicities**		
Cytopenia**^3^**	**n = 6**	**n = 9**
Grades I–IV	5 (83.3)	9 (100.0)
Grades III–IV	3 (50.0)	5 (55.3)
Anemia**^3^**	**n = 10**	**n = 10**
Grades I–IV	8 (80.0)	8 (80.0)
Grades III–IV	9 (90.0)	10 (100.0)
Leukopenia**^3^**	**n = 12**	**n = 12**
Grades I–IV	10 (83.3)	9 (75.0)
Grades III–IV	11 (91.7)	10 (83.3)
Thrombocytopenia**^3^**	**n = 14**	**n = 12**
Grades I–IV	12 (85.7)	9 (75.0)
Grades III–IV	11 (78.6)	11 (91.7)
**Infections^4^**	**n = 14**	**n = 18**
Total	12 (85.7)	16 (88.9)
Viral	8 (57.1)	9 (50.0)
Bacterial	5 (35.7)	8 (44.4)
Fungal	4 (28.6)	7 (38.9)
**OS^5^**	**n = 7**	**n = 12**
6 months	7 (100.0)	3 (25.0)
1 year	–	6 (50.0)
2 years	–	6 (50.0)

CRR, complete response rate; NA, not available; ORR, overall response rate; OS, overall survival; RCT, randomized controlled trials; SR-aGVHD, steroid-refractory acute graft-versus-host disease; SR-cGVHD, steroid-refractory chronic graft-versus-host disease.

**^1^** Twelve studies included both in the SR-aGVHD and SR-cGVHD analysis;

**^2^** Twenty-one studies involved multiple organs in the analysis of ORRs;

Eighteen studies involved multiple organs in the analysis of CRRs.

**^3^** Seven studies included both in the analysis of overall (grades I–IV) and severe (grades III–IV) cytopenia;

Fifteen studies included both in the analysis of overall (grades I–IV) and severe (grades III–IV) anemia;

Sixteen studies included both in the analysis of overall (grades I–IV) and severe (grades III–IV) leukopenia;

Seventeen studies included both in the analysis of overall (grades I–IV) and severe (grades III–IV) thrombocytopenia.

**^4^** Fourteen studies involved in the analysis of multiple types of infection.

**^5^** Three studies included both in the analysis of 6-month, 1-year, and 2-year OS in SR-cGVHD.

The bold values represent the total number of studies included in the analysis.

### Response rate

#### SR-aGVHD

##### ORR after ruxolitinib treatment

**Figure 2:**
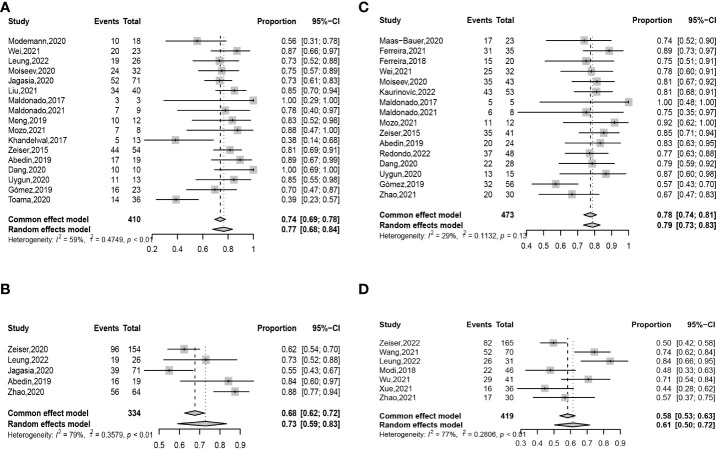
Forest plots of ORRs at any time **(A)** and day 28 **(B)** after ruxolitinib treatment in SR-aGVHD and the ORRs at any time **(C)** and week 24 **(D)** after ruxolitinib treatment in SR-cGVHD.

**Figure 3:**
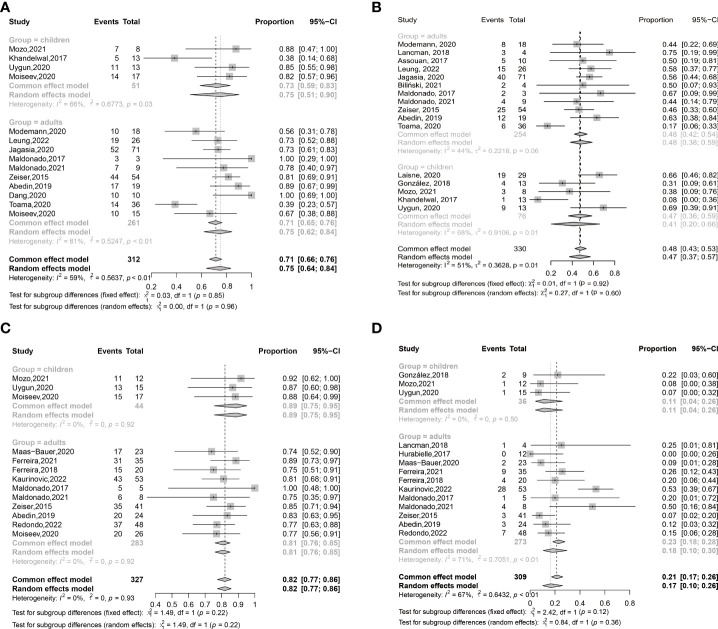
The subgroup analysis of adults and children in ORRs **(A)** and CRRs **(B)** at any time after ruxolitinib treatment in SR-aGVHD; The subgroup analysis of adults and children in ORRs **(C)** and CRRs **(D)** at any time after ruxolitinib treatment in SR-cGVHD.

ORR at any time after ruxolitinib treatment was 0.77 (95% confidence interval (CI): 0.68–0.84) (**Figure 2A**). ORR at any time was 0.78 (95% CI: 0.67–0.86) in retrospective studies and was 0.74 (95% CI: 0.64–0.81) in prospective unrandomized studies (**Figures S1A, B**). In adults, ORR at any time was 0.75 (95% CI: 0.62–0.84), which was comparable with that in children (0.75 (95% CI: 0.51–0.90), *P* = 0.960) (**Table S1**, **Table 6**, and **Figure 3A**).

**Table 6:** The summary table of comparisons between adults and children.

Subgroup	Adults		Children		*P*-value
	Cumulative incidence	95% CI	Cumulative incidence	95% CI	
**SR-aGVHD**					
ORR					
At any time	0.75	0.62–0.84	0.75	0.51–0.90	0.960
CRR					
At any time	0.48	0.42–0.54	0.41	0.20–0.66	0.601
At day 28	0.32	0.24–0.41	0.21	NA	NA
Infection	0.75	0.66–0.82	0.86	0.64–0.95	0.296
Viral infection	0.59	0.59–0.71	0.45	0.31–0.60	0.193
Cytopenia					
Grades I–IV	37.3	0.0–82.1	53.8	NA	NA
Anemia					
Grades I–IV	24.7	0.0–55.3	25.0	NA	NA
Grades III–IV	26.0	7.0–45.0	25.0	NA	NA
Leukopenia					
Grades I–IV	34.3	0.6–68.1	26.0	0.0–100.0	0.645
Grades III–IV	28.0	14.8–41.2	26.0	0.0–100.0	0.904
Thrombocytopenia					
Grades I–IV	23.7	0.0–56.7	19.8	0.0–41.8	0.722
Grades III–IV	31.0	0.0–73.2	30.5	0.0–100.0	0.975
**SR-cGVHD**					
ORR					
At any time	0.81	0.76–0.85	0.89	0.75–0.95	0.222
CRR					
At any time	0.18	0.10-0.30	0.11	0.04 –0.26	0.359
Infection	0.37	0.18–0.61	0.42	NA	NA
Anemia					
Grades I–IV	20.7	0.0–57.9	8.3	NA	NA
Grades III–IV	5.0	0.0–15.6	0.0	NA	NA
Leukopenia					
Grades I–IV	11.5	2.6–20.4	8.3	NA	NA
Grades III–IV	9.8	2.3–17.3	0.0	NA	NA
Thrombocytopenia					
Grades I–IV	7.0	0.0–17.6	8.3	NA	NA
Grades III–IV	3.8	0.0–8.3	0.0	NA	NA

ORR at day 28 after ruxolitinib treatment was 0.73 (95% CI: 0.59–0.83) (**Figure 2B**). ORRs at day 28 were 0.78 (95% CI: 0.63–0.88) and 0.74 (95% CI: 0.45–0.91), respectively, in retrospective studies and prospective unrandomized studies (**Figures S1C, D**). In the randomized controlled trial (RCT), ORR at day 28 was 0.62.

##### CRR after ruxolitinib treatment

**Figure 4:**
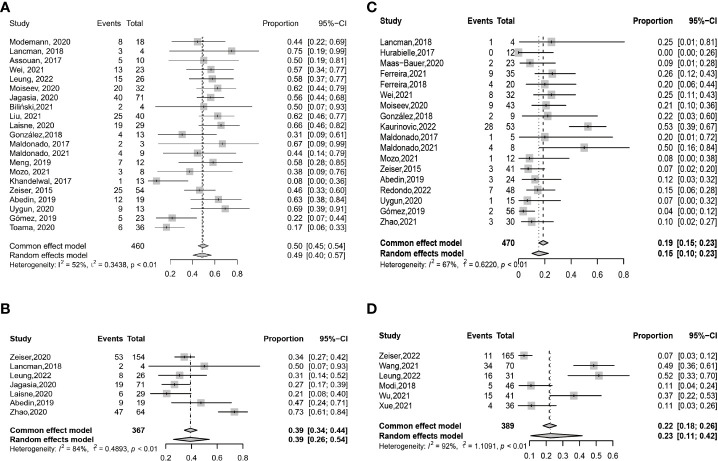
Forest plots of CRRs at any time **(A)** and day 28 **(B)** after ruxolitinib treatment in SR-aGVHD and the CRRs at any time **(C)** and week 24 **(D)** after ruxolitinib treatment in SR-cGVHD.

CRR at any time after ruxolitinib treatment was 0.49 (95% CI: 0.40–0.57) (**Figure 4A**). CRRs at any time were 0.48 (95% CI: 0.38–0.58) and 0.55 (95% CI: 0.46–0.64), respectively, in retrospective studies and prospective unrandomized studies (**Figures S3A, B**). CRRs at any time were comparable between adults and children (0.48 (95% CI: 0.42–0.54) vs. 0.41 (95% CI: 0.20–0.66), *P* =0.601) (**Table S1** and **Figure 3B**).

CRR at day 28 after ruxolitinib treatment was 0.39 (95% CI: 0.26–0.54) (**Figure 4B**). CRRs at day 28 were 0.32 (95% CI: 0.23–0.43) and 0.50 (95% CI: 0.19–0.81), respectively, in retrospective studies and prospective unrandomized studies (**Figures S3C, D**). In the RCT, CRR at day 28 was 0.34. CRRs at day 28 were 0.32 (95% CI: 0.24–0.41) and 0.21, respectively, for adults and children.

#### SR-cGVHD

##### ORR after ruxolitinib treatment

ORR at any time after ruxolitinib treatment was 0.78 (95% CI: 0.74–0.81) (**Figure 2C**). ORRs at any time were 0.77 (95% CI: 0.73–0.81) and 0.81, respectively, in retrospective studies and prospective unrandomized studies (**Figure S2A**). In adults, ORR at any time was 0.81 (95% CI: 0.76–0.85), which was comparable with that in children (0.89 (95% CI: 0.75–0.95), *P* =0.222) (**Table S1** and **Figure 3C**).

ORR at week 24 after ruxolitinib treatment was 0.61 (95% CI: 0.50–0.72) (**Figure 2D**). In retrospective studies, ORR at week 24 was 0.64 (95% CI: 0.51–0.75) (**Figure S2B**). In the RCT, ORR at week 24 was 0.50.

##### CRR after ruxolitinib treatment

CRR at any time after ruxolitinib treatment was 0.15 (95% CI: 0.10–0.23) (**Figure 4C**). CRR at any time was 0.15 (95% CI: 0.09–0.23) and 0.21 (95% CI: 0.12–0.34), respectively, in retrospective studies and prospective unrandomized studies, respectively (**Figures S4A, B**). In adults, CRR at any time was 0.18 (95% CI: 0.10-0.30), which was compared with that in children (0.11 (95% CI: 0.04 –0.26), *P* = 0.359) (**Figure 3D**).

CRR at week 24 after ruxolitinib treatment was 0.23 (95% CI: 0.11–0.42) (**Figure 4D**). In retrospective studies, CRR at week 24 was 0.29 (95% CI: 0.15–0.48) (**Figure S4C**). In the RCT, CRR at week 24 was 0.07.

### Response according to the involved organs after ruxolitinib treatment

#### SR-aGVHD

The ORRs and CRRs at any time after treatment were showed in **Table 7**. The CRRs at any time after treatment were highest in skin SR-aGVHD, followed by gut, and liver SR-aGVHD.

**Table 7:** Response at any time according to the involved organs after ruxolitinib treatment.

Subgroup	ORR		CRR	
	Cumulative incidence (%)	95%CI	Cumulative incidence (%)	95%CI
**SR-aGVHD**				
Skin	78.3	63.2–93.3	68.6	37.2–99.9
Gut	78.9	66.6–91.2	57.9	37.8–78.1
Liver	60.4	37.2–83.5	49.1	32.8–65.5
**SR-cGVHD**				
Skin	73.2	58.7–87.7	30.1	18.2–42.0
Gut	69.2	50.9–87.5	25.7	2.4–48.9
Liver	65.7	45.0–86.3	32.7	15.8–49.6
Mouth	76.5	61.5–91.5	34.0	24.7–43.3
Eyes	61.1	38.7–83.5	16.7	2.4–31.0
Lung	47.3	29.8–64.9	11.1	1.2–21.0
Joints and fascia	67.4	46.4–88.3	11.9	0.0–23.8
Esophagus	50.0	NA	0.0	NA

CI, confidence interval; CRR, complete response rate; NA, not available; ORR, overall response rate; SR-aGVHD, steroid-refractory acute graft-versus-host disease; SR-cGVHD, steroid-refractory chronic graft-versus-host disease.

#### SR-cGVHD

The ORRs at any time after treatment were highest in mouth SR-cGVHD, followed by skin and gut SR-cGVHD. The CRRs at any time after treatment was highest in mouth SR-cGVHD, followed by liver and skin SR-cGVHD (**Table 7**).

### Infections after ruxolitinib treatment

#### SR-aGVHD

**Figure 5:**
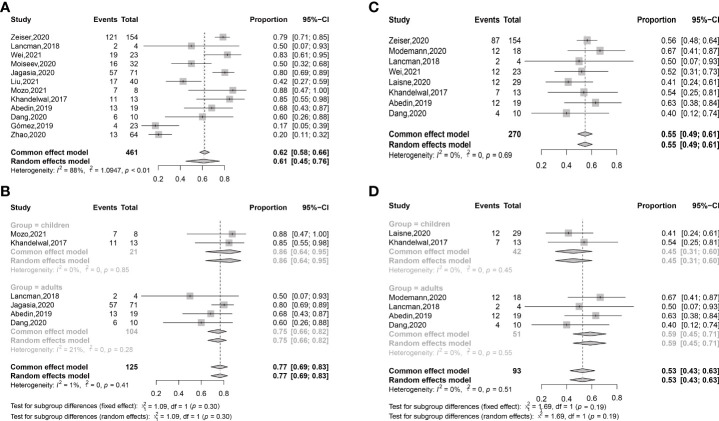
Forest plots of frequency rates of infections **(A)** and the subgroup analysis of adults and children **(B)** after ruxolitinib treatment in SR-aGVHD; Forest plots of frequency rates of viral infections **(C)** and the subgroup analysis of adults and children **(D)** after ruxolitinib treatment in SR-aGVHD.

The incidence rate of infections after ruxolitinib treatment was 0.61 (95% CI: 0.45–0.76). The frequency rates of infection after ruxolitinib treatment were comparable between children [0.86 (95% CI: 0.64–0.95)] and adults [0.75 (95% CI: 0.66–0.82), *P* = 0.296] (**Figures 5A, B**). The frequency rates of viral infections were 0.55 (95% CI: 0.49–0.61). The frequency rates of viral infection after ruxolitinib treatment were comparable between children [0.45 (95% CI: 0.31–0.60)] and adults [0.59 (95% CI: 0.59–0.71), *P* = 0.193] (**Figures 5C, D**).

#### SR-cGVHD

The incidence rate of infection after ruxolitinib treatment was 0.47 (95% CI: 0.31–0.63) (**Figure S5**). The frequency rates of infection after ruxolitinib treatment were 0.37 (95% CI: 0.18–0.61) and 0.42, respectively, for adults and children. The frequency rates of viral infection were 0.29 (95% CI: 0.25–0.34) (**Figure S6**).

### Myelosuppression after ruxolitinib treatment

#### SR-aGVHD

The incidence rates of overall (grades I–IV) and severe (grades III–IV) cytopenia were 53.2% (95% CI: 16.0%–90.4%) and 31.0% (95% CI: 0.0%–100.0%), respectively. The incidence rate of overall cytopenia was 37.3% (95% CI: 0.0–82.1%) and 54.0%, respectively, for adults and children. The frequency rates of anemia, leukopenia, and thrombocytopenia were 45.6% (95% CI:19.8%–71.5%), 44.2% (95% CI: 24.4%–64.0%), and 40.6% (95% CI: 21.4%–59.8%), respectively (**Table 8**).

**Table 8:** Myelosuppression after ruxolitinib treatment.

Subgroup	Total		Adults		Children	
	Cumulative incidence	95% CI	Cumulative incidence	95% CI	Cumulative incidence	95% CI
**SR-aGVHD**						
Cytopenia						
Grades I–IV	53.2	16.0–90.4	37.3	0.0–82.1	53.8	NA
Grades III–IV	31.0	0.0–100.0	16.5	0.0–100.0	NA	NA
Anemia						
Grades I–IV	45.6	19.8–71.5	24.7	0.0–55.3	25.0	NA
Grades III–IV	37.3	18.4–56.3	26.0	7.0-45.0	25.0	NA
Leukopenia						
Grades I–IV	44.2	24.4–64.0	34.3	0.6–68.1	26.0	0.0–100.0
Grades III–IV	36.8	20.7–52.9	28.0	14.8–41.2	26.0	0.0–100.0
Thrombocytopenia						
Grades I–IV	40.6	21.4–59.8	23.7	0.0–56.7	19.8	0.0–41.8
Grades III–IV	42.4	25.0–59.7	31.0	0.0–73.2	30.5	0.0–100.0
**SR-cGVHD**						
Cytopenia						
Grades I–IV	28.8	13.0–44.6	28.3	4.8–51.7	NA	NA
Grades III–IV	10.4	0.0–27.9	21.0	0.0–100.0	NA	NA
Anemia						
Grades I–IV	35.1	13.2–57.0	20.7	0.0–57.9	8.3	NA
Grades III–IV	11.2	2.1–20.3	5.0	0.0–15.6	0.0	NA
Leukopenia						
Grades I–IV	22.9	6.2–39.6	11.5	2.6–20.4	8.3	NA
Grades III–IV	8.9	4.7–13.1	9.8	2.3–17.3	0.0	NA
Thrombocytopenia						
Grades I–IV	19.2	6.9–31.6	7.0	0.0–17.6	8.3	NA
Grades III–IV	10.2	3.6–16.8	3.8	0.0–8.3	0.0	NA

CI, confidence interval; NA, not available; SR-aGVHD, steroid-refractory acute graft-versus-host disease; SR-cGVHD, steroid-refractory chronic graft-versus-host disease.

#### SR-cGVHD

The incidence rates of overall and severe cytopenia were 28.8% (95% CI: 13.0%–44.6%) and 10.4% (95% CI: 0.0–27.9%), respectively. The frequency rates of anemia, leukopenia, and thrombocytopenia were 35.1% (95% CI: 13.2%–57.0%), 22.9% (95% CI: 6.2%–39.6%), and 19.2% (95% CI: 6.9%–31.6%), respectively (**Table 8**).

### OS

#### SR-aGVHD

The probability rate of OS at 6 months after treatment was 63.9% (95% CI: 52.5%–75.2%). The probability rates of OS at 6 months after treatment were 65.6% (95% CI: 49.1%–82.1%) and 59.5% (95% CI: 0.0%–100.0%), respectively, for retrospective studies and prospective unrandomized studies.

#### SR-cGVHD

The probability rates of OS at 6 months, 1 year, and 2 years after treatment were 95% (95% CI: 79.5%–100.0%), 78.7% (95% CI: 67.2%–90.1%), and 75.3% (95% CI: 68.0%–82.7%), respectively.

## Discussion

Many studies have reported that ruxolitinib was effective treatment for patients with SR-GVHD, and we also observed that therapeutic response and survival seemed to be comparable between adults and children. To the best of our knowledge, this study is the most comprehensive systematic review to summarize the published studies and demonstrated the efficacy and safety of ruxolitinib treatment for SR-GVHD.

For SR-aGVHD, the ORRs of ruxolitinib at any time and at day 28 were 0.77 and 0.73, respectively. Many other therapeutic modalities were also applied to control SR-aGVHD. Prior data reported that the ORRs of antithymocyte globulin [ATG, (11, 38, 39)], extracorporeal photopheresis [ECP, (40–44)], mycophenolate mofetil [MMF, (45–50)], etanercept (51–55), daclizumab (56), inolimomab (56), and denileukin diftitox (56) were 0.30–0.31, 0.66–0.75, 0.31–0.67, 0.46–0.68, 0.71, 0.54, and 0.56, respectively. In addition, the ORR at day 28 after treatment was 0.54–0.56 for ATG (57, 58), and the ORRs at 1 month after treatment were 0.69, 0.55, and 0.56, respectively, for daclizumab (56), inolimomab (56), and denileukin diftitox (56). Thus, it seems that ruxolitinib has a higher ORR compared with most of the other second-line treatments in SR-aGVHD.

In this study, the CRRs of ruxolitinib at any time and at day 28 were 0.49 and 0.39, respectively, in patients with SR-aGVHD. The available data about the CRRs of ATG (11, 38, 39), ECP (40–44, 59–61), MMF (45, 46, 48, 50), etanercept (51, 52, 54, 55), mesenchymal stem cell (MSC) (62), daclizumab (56), inolimomab (56), and denileukin diftitox (56) were 0.08–0.14, 0.52–0.75, 0–0.31, 0–0.31, 0.09–0.65, 0.42, 0.30, and 0.37, respectively. In addition, the CRR at day 28 after treatment was 0.20–0.36 for ATG (57, 58), and the CRRs at 1 month after treatment were 0.37, 0.31, and 0.37, respectively, for daclizumab, inolimomab, and denileukin diftitox (56). Thus, the CRR of ruxolitinib did not seem to be more superior to other second-line treatments, which can be further improved.

For SR-cGVHD, the ORR of ruxolitinib at any time was 0.78. On the basis of data from previous systematic reviews, the ORRs of rituximab (63, 64), MMF (64), imatinib (64), MSC (64), methotrexate (64), ECP (43, 64), pentostatin (64), sirolimus (64), and thalidomide (64) were 0.66–0.68, 0.65, 0.58, 0.65, 0.70, 0.64–0.68, 0.54, 0.79, and 0.50, respectively. Ruxolitinib showed a higher ORR compared with most of the other second-line treatments in patients with SR-cGVHD.

Four studies reported that ruxolitinib could be used in the treatment for children with SR-GVHD (29–32); however, few studies compared the clinical outcomes of ruxolitinib between children and adults with SR-GVHD. In this study, we first observed that ORR, CRR, infection events, and myelosuppression were all comparable between adult and children, which suggested that ruxolitinib treatment was effective and safe for children with SR-GVHD.

In recently real-world studies, the ORR and CRR of basiliximab at day 28 were 0.66–0.79 and 0.52–0.61, respectively in patients with SR-aGVHD (65, 66). Compared with this study, the efficacy of basiliximab seemed to be compared with ruxolitinib. However, in the only successful RCT (REACH2 study) for SR-aGVHD, nine second-line treatments except interleukin-2 receptor antagonists were included as the best available treatments. Thus, comparing the safety and efficacy between ruxolitinib and basiliximab in SR-aGVHD seems to be an interesting clinical issue.

This study has several limitations. First, we observed that the ORR for ruxolitinib seemed to be superior to other drugs in SR-aGVHD and SR-cGVHD. However, differences existed in the patient selection or publication bias may influence the comparison between ruxolitinib and other drugs. Considering that most studies about SR-GVHD were single-arm–designed, we admitted that the comparison of ruxolitinib and other second-line therapies might be insufficient, and it is premature to conclude that ruxolitinib was superior to other drugs based on the results of our meta-analysis. REACH 2 and REACH 3 trials had observed that ruxolitinib was superior to most of the other second-line drugs in RCTs, and real-world analysis could help to further compare the efficacy and safety between ruxolitinib and other drugs in future. Second, the reducing accuracy of our result might due to heterogeneity of different studies in our analysis. Third, the sample of children was still relatively small, and the comparisons of efficacy and safety between adults and children were insufficient, which should be further identified in future.

## Conclusion

In summary, this study suggests that ruxolitinib is an effective and safe treatment for SR-GVHD, and both children and adults with SR-GVHD could benefit from ruxolitinib treatment.

## Data availability statement

The raw data supporting the conclusions of this article will be made available by the authors, without undue reservation.

## Author contributions

X-DM and M-ZS designed the study. SF, W-XH, and YY conducted data collection. M-ZS, SF, W-XH, and X-DM conducted data analysis and drafted manuscript. All authors contributed to the article and approved the submitted version.

## Funding

This work was supported by the Foundation for Innovative Research Groups of the National Natural Science Foundation of China (grant number 81621001), the Program of the National Natural Science Foundation of China (grant number 82170208), CAMS Innovation Fund for Medical Sciences (CIFMS) (grant number 2019-I2M-5-034), the Key Program of the National Natural Science Foundation of China (grant number 81930004), and the Fundamental Research Funds for the Central Universities.

## Conflict of interest

The authors declare that the research was conducted in the absence of any commercial or financial relationships that could be construed as a potential conflict of interest.

## Publisher’s note

All claims expressed in this article are solely those of the authors and do not necessarily represent those of their affiliated organizations, or those of the publisher, the editors and the reviewers. Any product that may be evaluated in this article, or claim that may be made by its manufacturer, is not guaranteed or endorsed by the publisher.
