# Supplementary material for: Efficacy and safety of ruxolitinib in steroid-refractory graft-versus-host disease: A meta-analysis
Source: Front Immunol. 2022 Aug 4;13:954268. doi: 10.3389/fimmu.2022.954268 (PMC9386528; doi:10.3389/fimmu.2022.954268)

**Supplementary Appendix**

**Title: Efficacy and Safety of Ruxolitinib in Steroid-Refractory Graft-Versus-Host Disease: A Meta-Analysis.**

**Contents**

**Supplementary Tables**

[Supplementary Table 1. The number of studies included in the subgroup analysis of adults and children. 2](#_Toc105353773)

**Supplementary Figures**

[Supplementary Figure 1. Forest plots of ORRs at any time of SR-aGVHD in retrospective studies (A) and prospective unrandomized studies (B) and the ORRs at day 28 of SR-aGVHD in retrospective studies (C) and prospective unrandomized studies (D). 3](#_Toc105353774)

[Supplementary Figure 2. Forest plots of ORRs at any time (A) and at week 24 (B) of SR-cGVHD in retrospective studies. 4](#_Toc105353775)

[Supplementary Figure 3. Forest plots of CRRs at any time of SR-aGVHD in retrospective studies (A) and prospective unrandomized studies (B) and the CRRs at day 28 of SR-aGVHD in retrospective studies (C) and prospective unrandomized studies (D). 5](#_Toc105353776)

[Supplementary Figure 4. Forest plots of CRRs at any time of SR-cGVHD in retrospective studies (A) and prospective unrandomized studies (B) and the CRRs at week 24 of SR-cGVHD in retrospective studies (C). 6](#_Toc105353777)

[Supplementary Figure 5. Forest plots of frequencies of overall infection after ruxolitinib treatment in SR-cGVHD. 7](#_Toc105353778)

[Supplementary Figure 6. Forest plots of frequencies of overall viral infection after ruxolitinib treatment in SR-cGVHD. 8](#_Toc105353779)

# Supplementary Table 1. The number of studies included in the subgroup analysis of adults and children.

| **Subgroup** | **SR-aGVHD** | |  | **SR-cGVHD** | |
| --- | --- | --- | --- | --- | --- |
|  | **Adults** | **Children** |  | **Adults** | **Children** |
| ORR |  |  |  |  |  |
| At any time | 10 | 4 |  | 10 | 3 |
| CRR |  |  |  |  |  |
| At any time | 11 | 5 |  | 11 | 3 |
| At day 28 | 4 | 1 |  | NA | NA |
| Infection | 4 | 2 |  | 8 | 1 |
| Viral infection | 4 | 2 |  | NA | NA |
| Cytopenia |  |  |  |  |  |
| Grade I-IV | 3 | 1 |  | NA | NA |
| Anemia |  |  |  |  |  |
| Grade I-IV | 3 | 1 |  | 3 | 1 |
| Grade III-IV | 4 | 1 |  | 4 | 1 |
| Leukopenia |  |  |  |  |  |
| Grade I-IV | 3 | 2 |  | 4 | 1 |
| Grade III-IV | 4 | 2 |  | 5 | 1 |
| Thrombocytopenia |  |  |  |  |  |
| Grade I-IV | 3 | 4 |  | 4 | 1 |
| Grade III-IV | 4 | 2 |  | 5 | 1 |
| CRR, complete response rate; NA, not available; ORR, overall response rate; SR-aGVHD, steroid-refractory acute graft-versus-host disease; SR-cGVHD, steroid-refractory chronic graft-versus-host disease. | | | | | |

# Supplementary Figure 1. Forest plots of ORRs at any time of SR-aGVHD in retrospective studies (A) and prospective unrandomized studies (B) and the ORRs at day 28 of SR-aGVHD in retrospective studies (C) and prospective unrandomized studies (D).


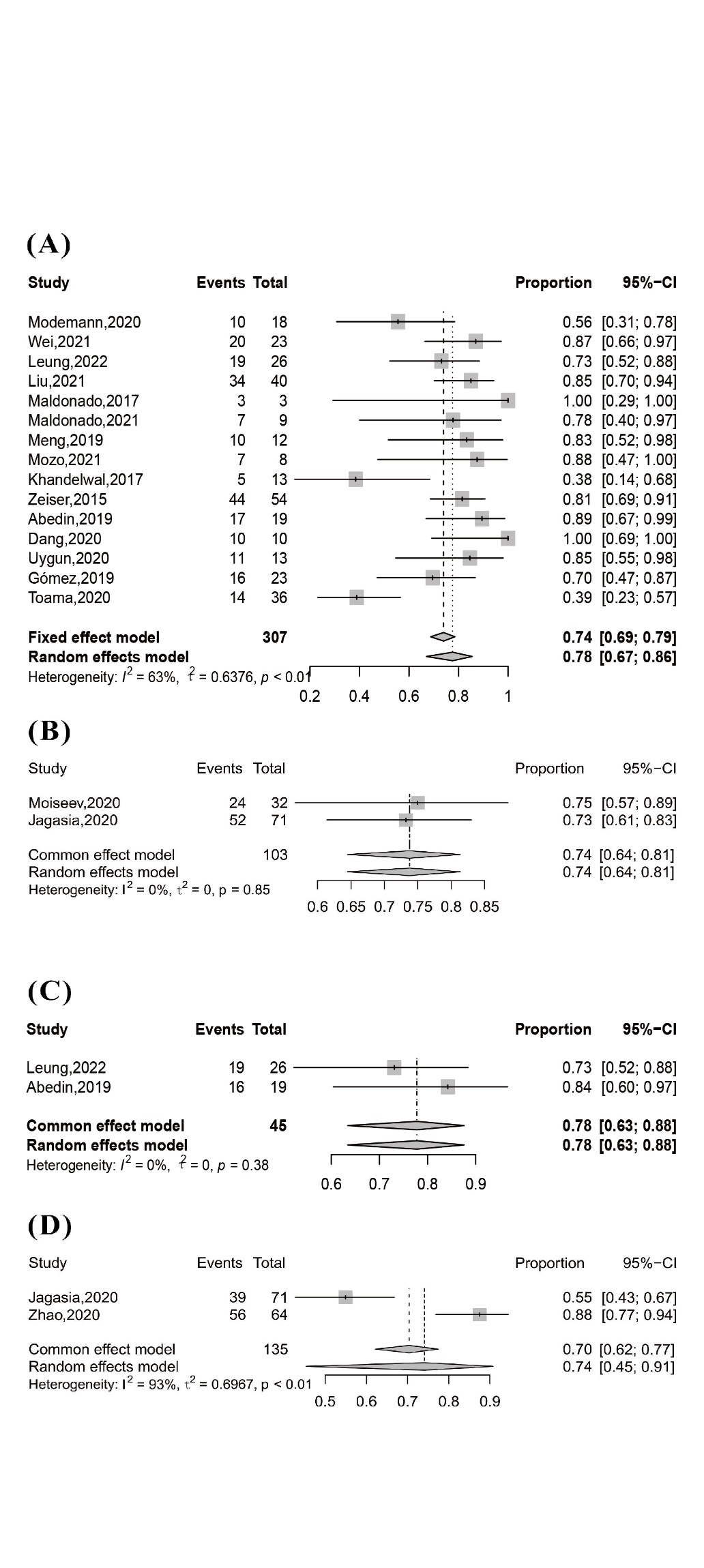


# Supplementary Figure 2. Forest plots of ORRs at any time (A) and at week 24 (B) of SR-cGVHD in retrospective studies.


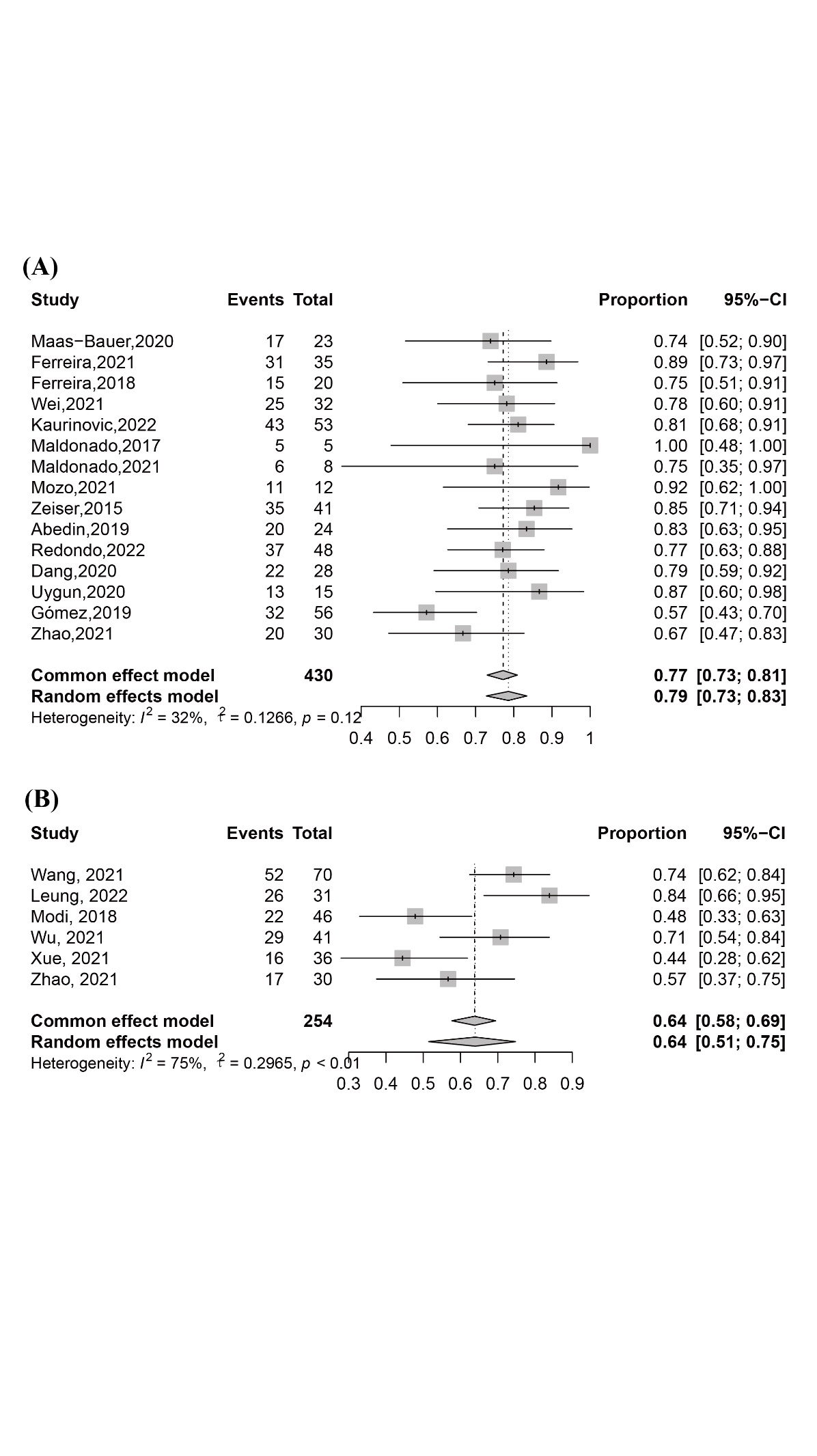


# Supplementary Figure 3. Forest plots of CRRs at any time of SR-aGVHD in retrospective studies (A) and prospective unrandomized studies (B) and the CRRs at day 28 of SR-aGVHD in retrospective studies (C) and prospective unrandomized studies (D).


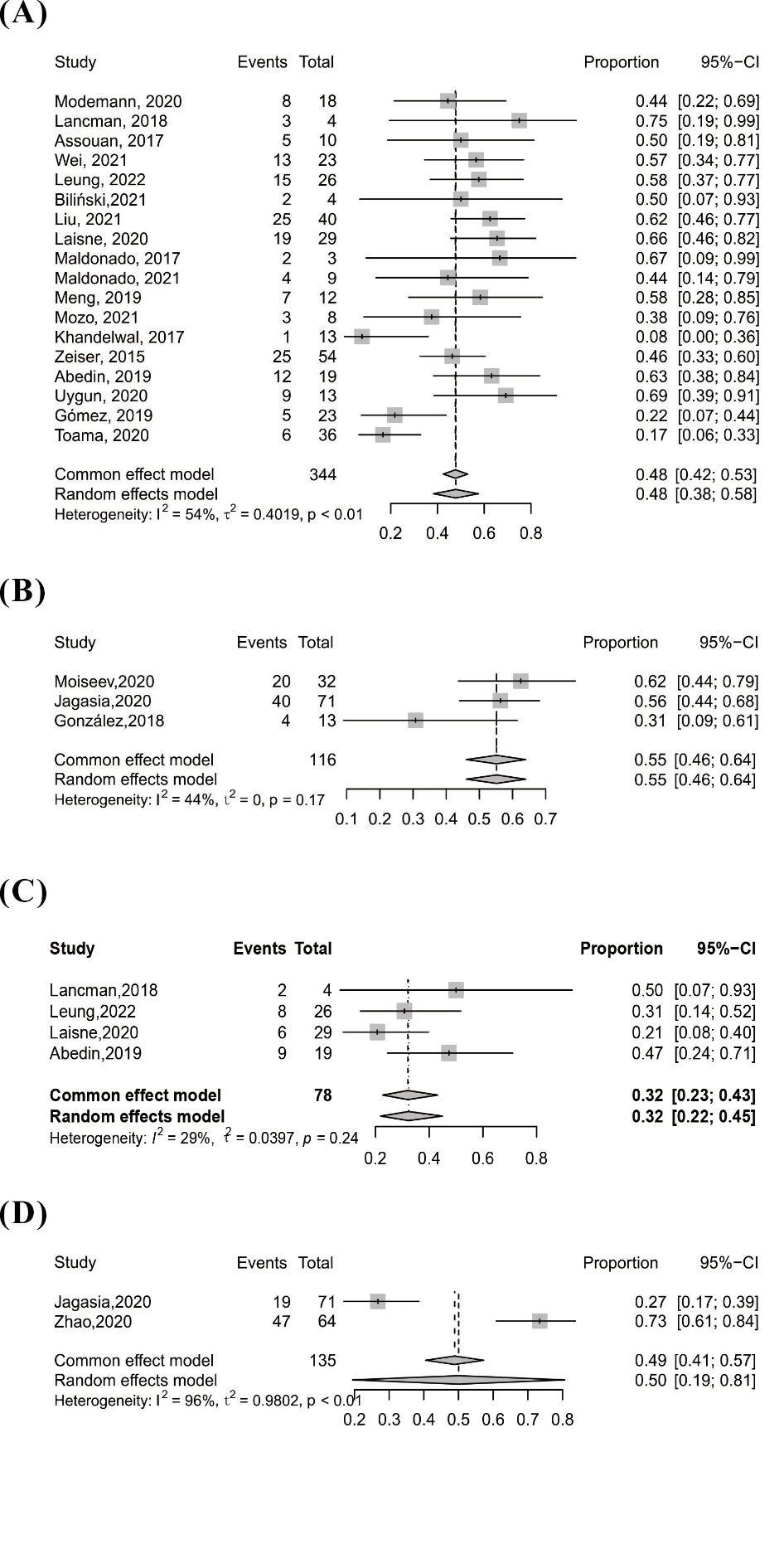


# Supplementary Figure 4. Forest plots of CRRs at any time of SR-cGVHD in retrospective studies (A) and prospective unrandomized studies (B) and the CRRs at week 24 of SR-cGVHD in retrospective studies (C).


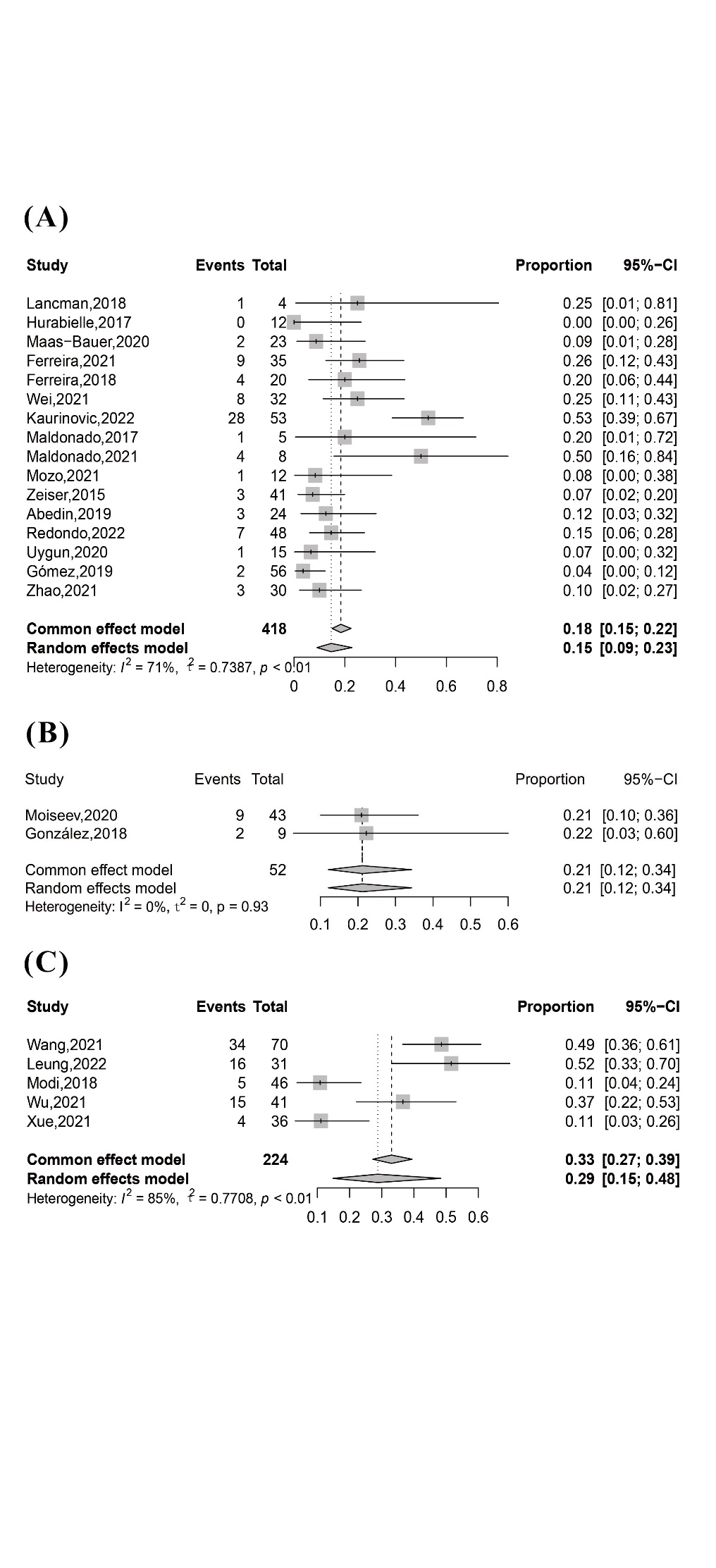


# Supplementary Figure 5. Forest plots of frequencies of overall infection after ruxolitinib treatment in SR-cGVHD.


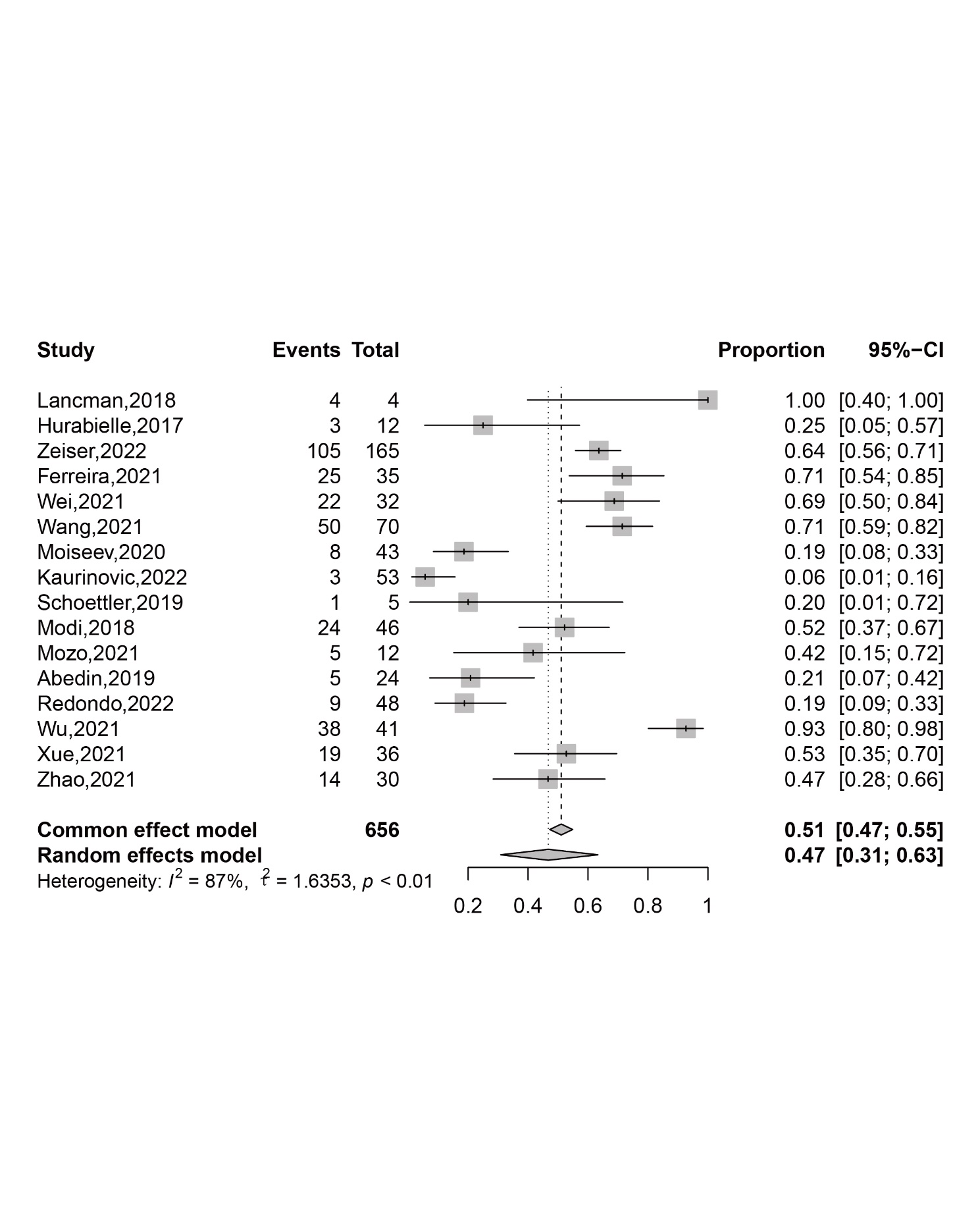


# Supplementary Figure 6. Forest plots of frequencies of overall viral infection after ruxolitinib treatment in SR-cGVHD.


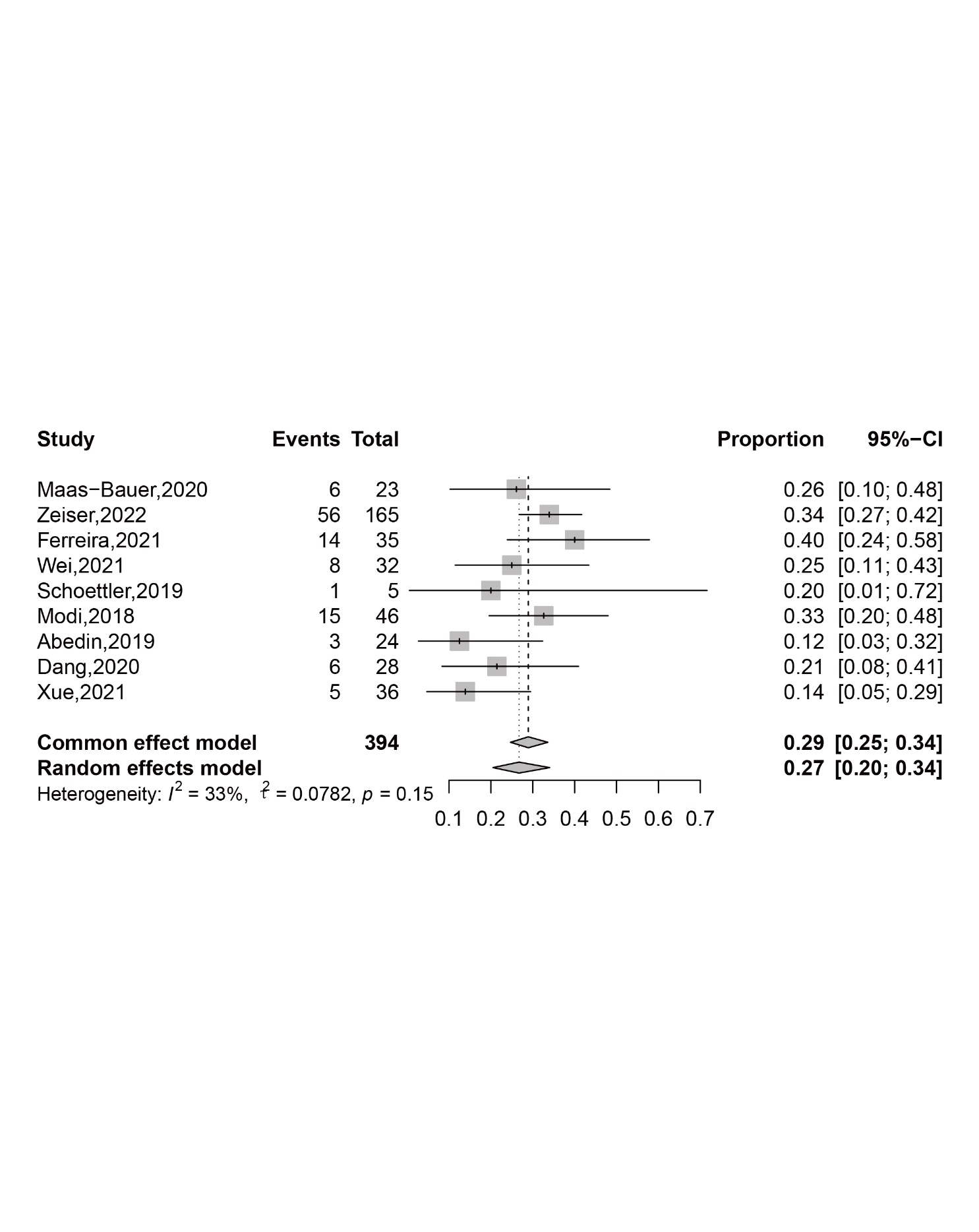

Supplement: Supplementary file 1 [file DataSheet_1.docx]
